# Proteome Analysis of *Camellia nitidissima* Chi Revealed Its Role in Colon Cancer Through the Apoptosis and Ferroptosis Pathway

**DOI:** 10.3389/fonc.2021.727130

**Published:** 2021-11-09

**Authors:** Yiwei Chen, Fan Zhang, Zhengcai Du, Jinling Xie, Lei Xia, Xiaotao Hou, Erwei Hao, Jiagang Deng

**Affiliations:** ^1^ School of Pharmacy, Chengdu University of Traditional Chinese Medicine, Chengdu, China; ^2^ Guangxi Key Laboratory of Efficacy Study on Chinese Materia Medica, Guangxi University of Chinese Medicine, Nanning, China; ^3^ Collaborative Innovation Center for Research on Functional Ingredients of Agricultural Residues, Guangxi University of Chinese Medicine, Nanning, China; ^4^ Postdoctoral Workstation, Guangxi Institute of Medicinal Plants, Nanning, China

**Keywords:** *Camellia nitidissima* Chi, colon cancer, ferroptosis, proteomics, 4D label-free

## Abstract

Colon cancer is the third most common cancer in the world with a high mortality rate. At present, surgery combined with radiotherapy and chemotherapy is the primary treatment, but patient prognosis remains poor. Traditional Chinese medicine (TCM) has become a complementary and alternative source of anti-cancer drugs. *Camellia nitidissima* Chi (CNC) is a TCM used to treat a variety of cancers. However, the role of CNC in cancer remains elusive, and its effect and mechanism on colon cancer have not been reported. Here, we show that CNC exerts an excellent inhibitory effect on colon cancer proliferation and apoptosis induction *in vitro* and *in vivo*. We performed label free-based quantitative proteomic analysis to evaluate the HCT116 cells treated with CNC. Our data revealed a total of 363 differentially expressed proteins, of which 157 were up-regulated and 206 down-regulated. Gene Ontology enrichment analysis showed that these proteins were involved in tumor occurrence and development through multiple biological processes such as cell proliferation, cell apoptosis, cell cycle, and cell death. Interestingly, we also found significant changes in ferroptosis pathways. The role of essential proteins glutathione peroxidase 4 (GPX4) and heme oxygenase-1 (HMOX1) were verified. CNC decreased the expression of GPX4 and increased the expression of HMOX1 at the mRNA and protein levels *in vivo* and *in vitro*. Collectively, these findings reveal that CNC regulates colon cancer progression *via* the ferroptosis pathway and could be an attractive treatment for colon cancer.

## Introduction

According to 2021 American Cancer Society data, colon cancer ranks third in terms of morbidity and mortality, and it is the third most common cancer in the world after lung and breast cancer (1). While surgery, chemotherapy, radiotherapy, and vaccine therapy can be applied individually or in combination, they carry side effects that affect patient quality of life (2, 3). This underscores the urgent need to develop novel strategies for the treatment of colon cancer.

Clinical studies have shown that traditional Chinese medicine (TCM) has significant advantages as auxiliary anti-tumor treatment. It has become a supplementary and alternative source of anti-cancer drugs with high efficiency and few side effects (4, 5). *Camellia nitidissima* Chi (CNC) is a TCM that mainly composed of flavonoids, saponins, and polyphenols (6). It has been used as a traditional folk medicine to treat hypertension, hyperlipidemia, infection, and other diseases (7). Moreover, a growing body of modern pharmacological research demonstrated that CNC has a significant inhibitory effect on various cancers (8–11). Despite, evidence supporting beneficial effects of CNC, its anti-cancer mechanisms remains unknown due to its multi-target effects and multi-component characteristics.

In recent years, proteomics research based on mass spectrometry (MS) has been widely used to investigate protein alterations (12, 13), and it is increasingly used to study TCM (14, 15). In the current study, we performed high-resolution MS to detect changes in crucial proteins in HCT116 cells treated with CNC. Bioinformatics analysis was subsequently carried out to identify novel pathways and protein targets affected by CNC treatment, and functional analysis was performed to predict the effect of molecular and biological processes to better understand the potential mechanism of CNC against colon cancer.

## Materials and Methods

### CNC Extract Preparation

In total, 400 g of dried CNC (harvested in Fangchenggang, Guangxi, China) was weighed, immersed in 10 L of 75% (v/v) ethanol for 30 min, and twice extracted in a reflux extraction for 1.5 h each time. The extracted solution was merged and evaporated by a rotary evaporator and then freeze-dried to obtain the extract powder. Dimethyl sulfoxide was dissolved into a 0.22-μM filter membrane with a concentration of 100 mg/mL for *in vitro* assays. Sterilized water was dissolved with a concentration of 1 g/mL (quantity of raw material) for *in vivo* assays and then frozen at -20°C.

#### Cell Lines and Animals

HCT116, SW480, and HCT15 cell lines (human colorectal adenocarcinoma) were obtained from the Type Culture Collection of the Chinese Academy of Sciences (Shanghai, China) and Procell Life Science &Technology Co., Ltd. (Wuhan, China). HCT116 was grown in McCoy’s 5A (Procell, China) supplemented with 10% fetal bovine serum (FBS; Gibco/Thermo Fisher Scientific, Waltham, MA, USA). SW480 was grown in L-15 media (Gibco/Thermo Fisher Scientific) supplemented with 10% FBS. HCT15 was grown in RPMI 1640 (Keygen, Nanjing, China) supplemented with 10% FBS. All cells were maintained at 37°C in a humidified incubator containing 5% CO_2_. Female BALB/c nude mice (5-6 weeks) purchased from Vital River (SCXK [Jing] 2016-0006, Beijing, China) were used for the *in vivo* experiments. Animals were housed at a controlled temperature of 20-22°C and relative humidity of 50-60% under12-h light-dark cycles.

#### Antibodies and Primer Sequences

The following antibodies were used: Caspase 3 (Cat# CST9661S), Caspase 9 (Cat# CST9507S), cyclin-dependent kinase (CDK4, Cat# 12790T), CDK6 (Cat# CST13331T), proliferating cell nuclear antigen (PCNA, Cat# CST13110T), glutathione peroxidase 4 (GPX4, Cat# CST52455S), heme oxygenase-1 (HMOX1, Cat# CST5853S), glyceraldehyde 3-phosphate dehydrogenase (GAPDH, Cat# CST5174S), all of which were from Cell Signaling Technology (CST, Danvers, MA, USA); Bax (Cat# ab77566) and Bcl-2 (Cat# ab59348) were from Abcam.

The primers used for the amplification of GPX4, HMOX1, P53, SLC7A11, FTH1, ACSL4 and GAPDH were synthesized by Generay Co., Ltd. (Shanghai, China). GPX4 sequence: forward primer (5'-3'), TTGCCGCCTACTGAAGC; reverse primer (5'-3'), ATGTGCCCGTCGATGTC. HMOX1 sequence: forward primer (5'-3'), CAGTCTTCGCCCCTGTCT; reverse primer (5'-3'), GCATGGCTGGTGTGTAGG. P53 sequence: forward primer (5'-3'), CAGCACATGACGGAGGTTGT; reverse primer (5'-3'), TCATCCAAATACTCCACACGC. SLC7A11 sequence: forward primer (5'-3'), TCTCCAAAGGAGGTTACCTGC; reverse primer (5'-3'), AGACTCCCCTCAGTAAAGTGAC. FTH1 sequence: forward primer (5'-3'), ATCTCATCAAGCCCTCTGTAGT; reverse primer (5'-3'), GGACGCAGGTCATGGAAGC. ACSL4 sequence: forward primer (5'-3'), ACTGGCCGACCTAAGGGAG; reverse primer (5'-3'), GCCAAAGGCAAGTAGCCAATA. GAPDH sequence: forward primer (5’-3’), GGACCTGACCTGCCGTCTAG; reverse primer (5’-3’), GTAGCCCAGGATGCCCTTGA.

### Analysis of Chemical Constituents in CNC Extract

Chromatographic analysis of CNC was performed using the UltiMate 3000 RS liquid chromatography (LC) system (Thermo Fisher Scientific). The gradient system consisting of acetonitrile containing 0.1% formic acid (solvent A) and water containing 0.1% formic acid (solvent B) was as follows: 0-1 min, 98% B; 1-25 min, 98-5% B; 25-30 min, 5-98% B. The volume of sample was 5 μL for each injection. The column temperature and flow rate were 35°C and 0.3 mL/min, respectively. CNC chemical constituents were detected by a Q Exactive Orbitrap mass spectrometer (Thermo Fisher Scientific) with an electrospray ionization interface. The full scan range was from 150 to 2000 m/z.

#### Cell Proliferation Assays and Morphological Observation

Cell viability was assessed with MTS kits according to the manufacturer instructions (Promega, Madison, WI, USA). Briefly, 3,000 cells/well were seeded in 96-well plates. After overnight incubation, the cells were treated with or without CNC extract (31.25, 46.875, 62.5, 93.75, 125, 187.5, 250 μg/mL) for 48 h prior to the MTS assays. The classic ferrostatin-1 rescue experiment was performed by MTS, the concentration of ferrostatin-1 was 1uM, and the concentration of CNC extract was 100ug/mL. Cells morphology was observed and photographed under an inverted microscope (Olympus, Tokyo, Japan).

#### Colony Formation Assays

To evaluate colony formation, 1,000 cells/well were seeded in 6-well plates. Then cells were treated with or without CNC extract (25, 50, 100 μg/mL). The plates were washed with phosphate-buffered saline (PBS) and stained with crystal violet on day 7. The cells were observed and photographed with the colony count analysis system of GelCount (Oxford Optronix, Abingdon, UK).

#### Apoptosis and Cell Cycle Assays

For apoptosis and cell cycle analyses, 1.5 × 10^5^ cells/well were seeded in 6-well plates. After overnight incubation, the cells were treated with or without CNC extract (25, 50, 100, 150 μg/mL) for 48 h. Cells were collected and performed according to the protocol of the Annexin V FITC Apoptosis kit (and FxCycleTM PI/RNAse Solution (both from BD Biosciences, Franklin Lakes, NJ, USA). All cell samples were analyzed on the Attune Flow cytometer (Thermo Fisher Scientific) within 1 h.

#### Western Blotting

The concentrations of HCT116 cells protein samples were determined using a Pierce™BCA Protein Assay Kit (Thermo Fisher Scientific). The protein samples were then separated by 12% SDS-PAGE (Bio-Rad, Hercules, CA, USA) and transferred to polyvinylidene fluoride membranes (Millipore, Burlington, MA, USA). After blocking for 1 h, antibodies were incubated overnight at 4°C. Protein bands were visualized utilizing ECL detection reagents (Bio-Rad).

### Proteomic Sample Preparation

HCT116 cell samples were disrupted in lysis buffer (1% Triton X-100, 1% protease inhibitor cocktail). Protein concentrations were determined by the BCA kit. 4D Label-free samples underwent reversed-phase peptide fractionation on a homemade rig followed by and LC-MS/MS analysis. Proteomic samples were analyzed by LC-MS/MS as described in **Supplement 1**.

### Protein Identification and Quantitation

LC-MS/MS spectra were searched using the MaxQuant search engine (v.1.6.6.0) (16) embedded into the Homo sapiens 9606 SwissProt database (20366 entries). Trypsin/P was specified as the cleavage enzyme allowing up to two missing cleavages. All identified proteins were subjected to the annotation methods as described in **Supplement 2**.

### Parallel Reaction Monitoring Analysis

Based on the method mentioned by Sun and colleagues (16), dithiothreitol and iodide acetamide were added to the protein solution. The mixture was hydrolyzed overnight at 37°C and the ratio of trypsin: protein was 1:50. Hydrolysis was continued for 4 h until the ratio of trypsin:protein was 1:100. The peptides were dissolved in 0.1% (v/v) aqueous formic acid, and the mobile phase was isolated using an EASY-nLC 1000 ultra high-performance LC (UHPLC) system (Thermo Fisher Scientific). The peptides were subjected to the nanospray ionization source followed by MS/MS in Q ExactiveTM Plus (Thermo Fisher Scientific) coupled online to the UHPLC. MS/MS data were processed using Skyline (v.3.6).

### Transmission Electron Microscopy

HCT116 and HCT15 cells were seeded into 15-cm dishes. After 24 h of culture, cells were treated with or without CNC extract (100μg/mL) for 48 h. Cells were collected and fixed, then dehydrated and embedded. The embedded cells were cut into ultrathin sections at 60-80nm using an ultrathin slicer (Leica UC7, Wetzlar, Germany). Finally, mitochondria ultrastructure was observed under TEM (HT7700, Hitachi, Tokyo, Japan) and the images were collected.

#### Detection of Reactive Oxygen Species, Iron, and Glutathione

HCT116 cells were treated with or without CNC extract for 12 h. Then, cells were collected and incubated with 10 μM 2’-7’-dichlorofluorescein diacetate (DCFH-DA) at 37°C for 25 min. The ROS-induced fluorescence intensity of intracellular DCFH-DA was measured by Operetta CLS™ High Content (PerkinElmer, Waltham, MAUSA). The iron concentrations in HCT116 and HCT15 were measured by Tissue iron assay kit. The glutathione (GSH) concentrations in HCT116 were measured by Reduced GSH assay kit.

### Reverse Transcription-Quantitative Polymerase Chain Reaction

According to the manufacturer’s instructions, total RNA was extracted using an Eastep R Super Total RNA Extraction Kit (Promega). cDNA was synthesized using a GoScript TM Reverse Transcription System (Promega). RT-QPCR was performed in 96-well plates using the Step-Two Real-Time PCR system (Applied Roche LightCycler 96). For quantitative SYBR^®^Green real-time PCR, the following primers were used: GPX4, HMOX1, P53, SLC7A11, FTH1, ACSL4 and GAPDH (all synthesized by Generay Co., Ltd.). GAPDH was used as an internal standard, and the relative expression of each gene was normalized to GAPDH. The relative quantification of gene expression was analyzed using the 2^-ΔΔCt^ method. Each sample was analyzed in triplicate.

### Immunofluorescence

Tumor specimens were fixed in 4% paraformaldehyde overnight and embedded in paraffin. Samples were incubated with primary antibodies against GPX4 (1:100, Proteintech), HMOX1 (1:100, Proteintech), CDK4 (1:300, CST), PCNA (1:600, CST), P53 (1:500, CST) or FTH1 in (1:300, CST) PBS overnight at 4°C. Samples were them incubated with secondary Cy3-labeled anti-rabbit antibody (Boster Biological Technology, Pleasanton, CA, USA) for 1 h at ambient temperature. DAPI was added and incubated for 5min in the dark. Samples were visualized with an Olympus BX53 fluorescence microscope.

### Subcutaneous Tumor Mouse Model

Nude mice were subcutaneously injected with 4×10^6^ HCT116-Luc cells. Mice with a tumor diameter of 100mm^3^ were numbered and randomly divided into five groups (n = 6/group). CNC extract (1.2, 2.4, or 4.8 g/kg/d) given *via* gavage. Fluorouracil (25mg/kg/2d) was injected intraperitoneally as the positive control. Tumor growth data were recorded twice a week, and the tumor volume was calculated. The mice were sacrificed and photographed after 14 days. The animal study protocol was reviewed and approved by the Animal Ethics Committee of the Guangxi University of Chinese Medicine, and all procedures were followed the relevant regulations and guidelines.

### Statistical Analysis

One-way analyses of variance followed by the LSD-t multiple comparison tests were performed to compare multiple groups. Each experiment was carried out in duplicate and repeated three times. Statistical analyses were performed using GraphPad 8.0.2 software (GraphPad Inc., San Diego, CA, USA) to perform a two-tailed t-test, and p < 0.05 was considered statistically significant. The results are presented as mean ± standard deviation (SD).

## Results

### Identification of Anti-tumor Active Compounds in CNC Extracts

We analyzed the phytochemical ingredients of CNC extract using UHPLC-MS. According to the standard of the mzCloud database (https://www.mzcloud.org/Stats), a total of 116 compounds with mzCloud best match >80 were identified **(see** **Supplementary 3** **)**. In addition, by further alignment with published data from the literature, 10 compounds were reported to have been isolated from CNC, and were speculated to be the anti-tumor active components. The details of these 10 compounds are listed in **Table 1**, including flavonoids, polysaccharides, volatile components, and triterpenoids. The base peak chromatogram of CNC extract obtained by UHPLC-MS was constructed (see **Supplementary 4** **)**.

**Table 1:** Relevant analytical data for compounds isolated from CNC extract.

Retention time (min)	Experimental mass (m/z)	Molecular formula	Theoretical mass (m/z)	Adduct ion	Mass error (ppm)	Possible compounds	Score
0.492	180.06369	C_6_H_12_O_6_	180.06339	[M+H]^+^	1.6661	D- (+)-Glucose	86.2
7.654	289.0722	C_15_H_14_O_6_	290.0793	[M−H]^−^	-0.7968	Catechin	93.6
10.119	609.1463	C_27_H_30_O_16_	610.1533	[M−H]^−^	0.0719	Rutin	95
11.411	433.1122	C_21_H_20_O_10_	432.1054	[M+H]^+^	0.5708	Vitexin	85.6
12.328	303.0497	C_15_H_10_O_7_	302.0424	[M+H]^+^	0.7157	Quercetin	88.3
12.822	287.0548	C_15_H_10_O_6_	286.0476	[M+H]^+^	0.5325	Luteolin	82.9
18.539	151.0391	C_8_H_8_O_3_	152.0463	[M−H]^−^	6.7101	Methyl salicylate	77.3
19.451	439.36035	C_30_H_48_O_3_	439.3563	[M+H]^+^	-2.8705	Oleanolic acid	94.8
20.124	427.3926	C_30_H_50_O	426.3856	[M+H]^+^	1.2408	Lupeol	86.8
21.471	283.2645	C_18_H_36_O_2_	284.2718	[M−H]^−^	-0.8516	Stearic acid	99.7

### CNC Extract Inhibits Cell Proliferation *In Vitro*

**Figure 1:**
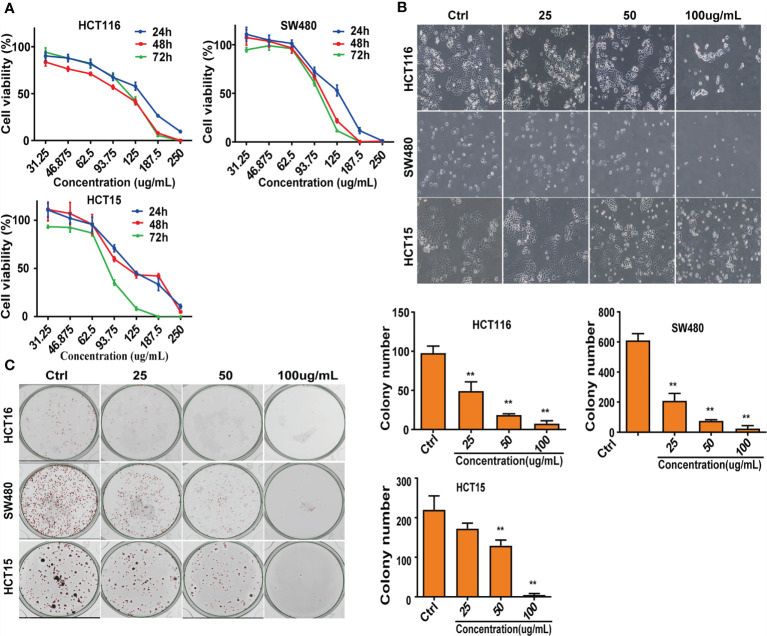
Suppress of cell lines growth *in vitro*. **(A)** After intervention with CNC extract, cell viability was measured by MTS assay. **(B)** Cell morphology were observed after treatment with CNC extract. **(C)** Number of cell clones after treatment with CNC extract. There were three duplicate samples in each experiment. Data are expressed as mean ± SD. Compared with control group: **p < 0.01.

**Figure 2:**
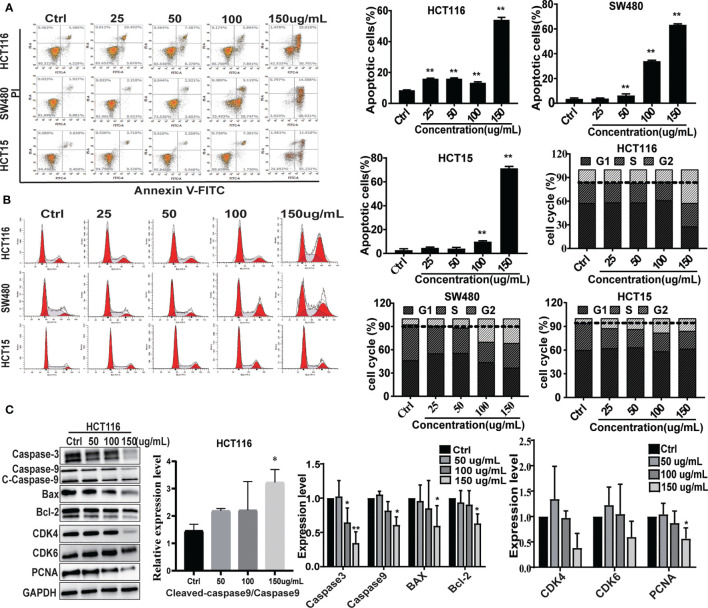
The effect on cell apoptosis and cell cycle. **(A)** Apoptosis rate induced by CNC extract in cell lines. **(B)** The cell cycle distribution of cell lines incubated by CNC extract. **(C)** WB analysis of related proteins in HCT116 after intervention of CNC extract. There were three duplicate samples in each experiment. Data are expressed as mean ± SD. Compared with control group: *p < 0.5, **p < 0.01.

To evaluate the effect of CNC extract on colon cancer cell growth, we treated HCT116, SW480, and HCT15 cells with different concentrations. All of the tested cell lines were treated with various concentrations of CNC extract for 24, 48, and 72h and had increasingly lower cell viability with higher drug concentrations **(** **Figure 1A** **)**. The half-maximal inhibitory concentrations (IC_50_) of CNC extract in HCT116, SW480, and HCT15 cells were 124.2μg/mL (24h), 92.37μg/mL (48h), 108.6μg/mL (72h), 124.2μg/mL (24h), 103.5μg/mL (48h), 97.98μg/mL (72h); and 128.5μg/mL (24h), 97.17μg/mL (48h), 84.38μg/mL (72h) respectively. Cell morphology gradually became round, and nuclear condensation induced by CNC extract was observed in all three cell lines **(** **Figure 1B** **)**. The colony formation abilities of HCT116, SW480, and HCT15 cells were dose-dependently inhibited after CNC extract incubation **(** **Figure 1C** **)**. The effects of different CNC extract concentration were evaluated by flow cytometry. Higher concentrations resulted in increased percentages of apoptotic cells in SW480 and HCT15. However, the apoptosis rate of HCT116 did not show a good dose-dependent concentration, perhaps because HCT116 was less sensitive, and the concentration of CNC extract that induces the initiation of mass apoptosis of HCT116 may be between 100 and 150μg/mL. **(** **Figure 2A** **)** and a greater proportion of G2/M phase cells **(** **Figure 2B** **)** compared to control. CNC extract interrupted cell cycle progression by inducing G2/M arrest rather than by influencing G0/G1. Moreover, we examined classical apoptosis proteins by western blot and found an increased ratio of cleaved-caspase-9/caspase-9 (C-caspase9/caspase9)were up-regulated after treated with CNC extract treatment, while caspase-3 was down-regulated **(** **Figure 2C** **)**. We also examined mitochondrial apoptosis proteins and found that although bcl-2 was down-regulated, the bax/bcl-2 ratio did not change significantly. These results may suggest that CNC extract induces apoptosis may through other signaling pathways. For cell cycle proteins, the results showed that CNC extract treatment decreased the levels of CDK4, 6, and PCNA in HCT116 cells.

### Quantitative Proteomics Analysis

**Figure 3:**
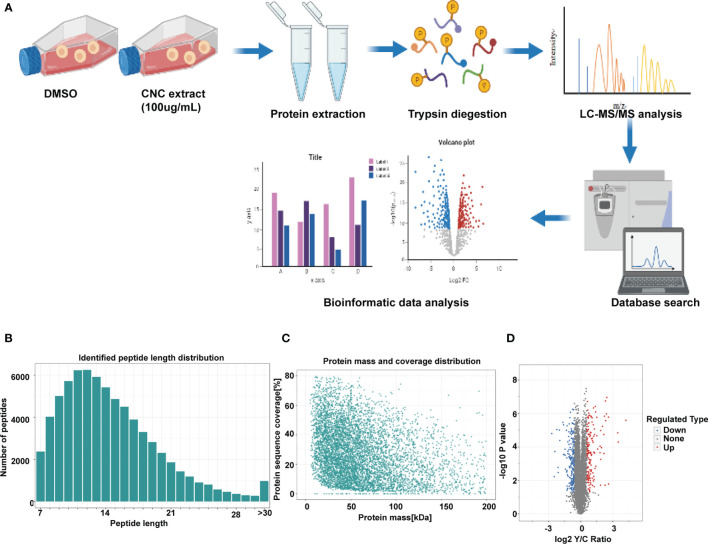
Differentially expressed proteins were evaluated by proteomics. **(A)** Flow diagram of 4D Label-free proteomics. **(B)** Length distribution of the peptides identified by mass spectrometry. **(C)** Relationship between protein molecular weight and coverage. **(D)** Volcano plot of differentially expressed proteins. The blue dots are down-regulated proteins. The red dots are up-regulated proteins. There were three duplicate samples in each experiment.

Proteomic data were acquired by 4D-Label free technology and a timsTOF pro mass spectrometer (Bruker nanoElute system, Billerica, MA, USA). Peptides digested by trypsin were analyzed using high-resolution MS. The primary ions and secondary fragments of the peptides were also subjected to high-resolution MS. All obtained data were analyzed through a specific bioinformatics database **(** **Figure 3A** **)**. Most peptide fragments were found to contain 7-20 amino acid residues, an observation that conforms with the general rules of trypsin enzymatic hydrolysis and higher-energy collisional dissociation fragmentation **(** **Figure 3B** **)**. There was a negative correlation between molecular weight and the protein sequence coverage. Larger molecular weight proteins can produce more enzymatic fragments to achieve the same coverage. That is to say. It needs more peptides identified from a large protein to achieve the same coverage. The length of the peptide fragments identified met the quality control requirements **(** **Figure 3C** **)**. In this experiment, we identified 6720 proteins, of which 6039 were quantified. Using 1.5-fold expression as the standard, we found that 363 proteins were differentially expressed in HCT116 cells treated with CNC extract. Among these, 157 and 206 proteins were up- and down-regulated, respectively. **(** **Figure 3D** **)**.

### Protein Function Classification and Enrichment Analysis

**Figure 4:**
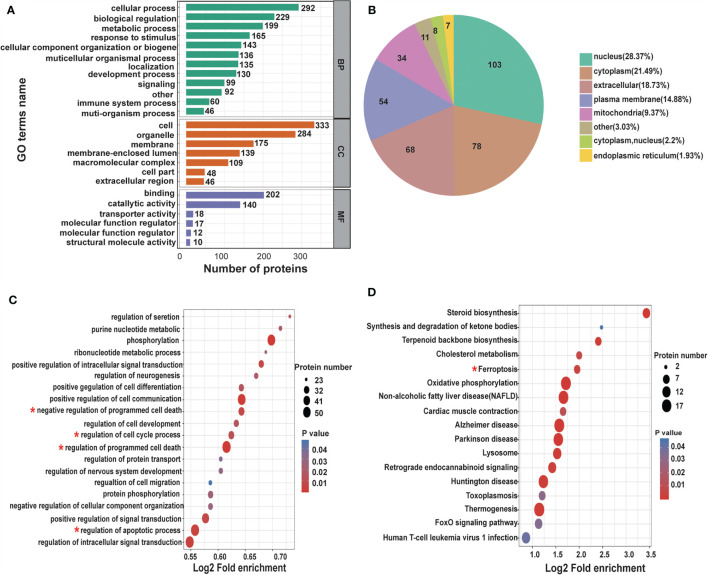
Differentially expressed proteins under the presence of CNC extract in HCT116 cells analyzed by GO and KEGG. **(A)** BP, CC, and MF evaluated. **(B)** Subcellular localization and classification of differentially expressed proteins. **(C)** GO enrichment analysis of BP. **(D)** KEGG pathway enrichment analysis.

To thoroughly understand the role of the differentially expressed proteins in the anti-tumor effect of CNC extract, we annotated the function and characteristics of these proteins from the Gene Ontology (GO) and Kyoto Encyclopedia of Genes and Genomics (KEGG). GO analysis indicated that the most two significantly changed biological processes (BP) were cellular process and biological regulation. Other functional categories such as cellular component (CC) and molecular function (MF)are listed in **Figure 4A**. InterProScan (http://www.ebi.ac.uk/interpro/) was utilized to analyze the subcellular classification of the differentially expressed proteins. Out of the 363 proteins, 139 are in the nucleus (28.31%), 119 are located in the cytoplasm (24.24%), and 82 are extracellular (16.7%) **(** **Figure 4B** **)**. In the GO enrichment analysis, multiple BPs such as regulation of cell proliferation, regulation of cell cycle, apoptotic process were involved in cancer development **(** **Figure 4C** **)**. The KEGG pathway enrichment analysis identified the ferroptosis pathway as being cancer related **(** **Figure 4D** **)**. Ferroptosis is a type of cell death characterized by elevated intracellular iron and ROS. The results suggest that CNC extract suppression of CC proliferation may be related to the ferroptosis pathway, and it might target GPX4 and HMOX1. In addition, it is associated with cell apoptosis and cell cycle arrest.

### CNC Extract Induces Cell Death by Promoting Ferroptosis

**Figure 5:**
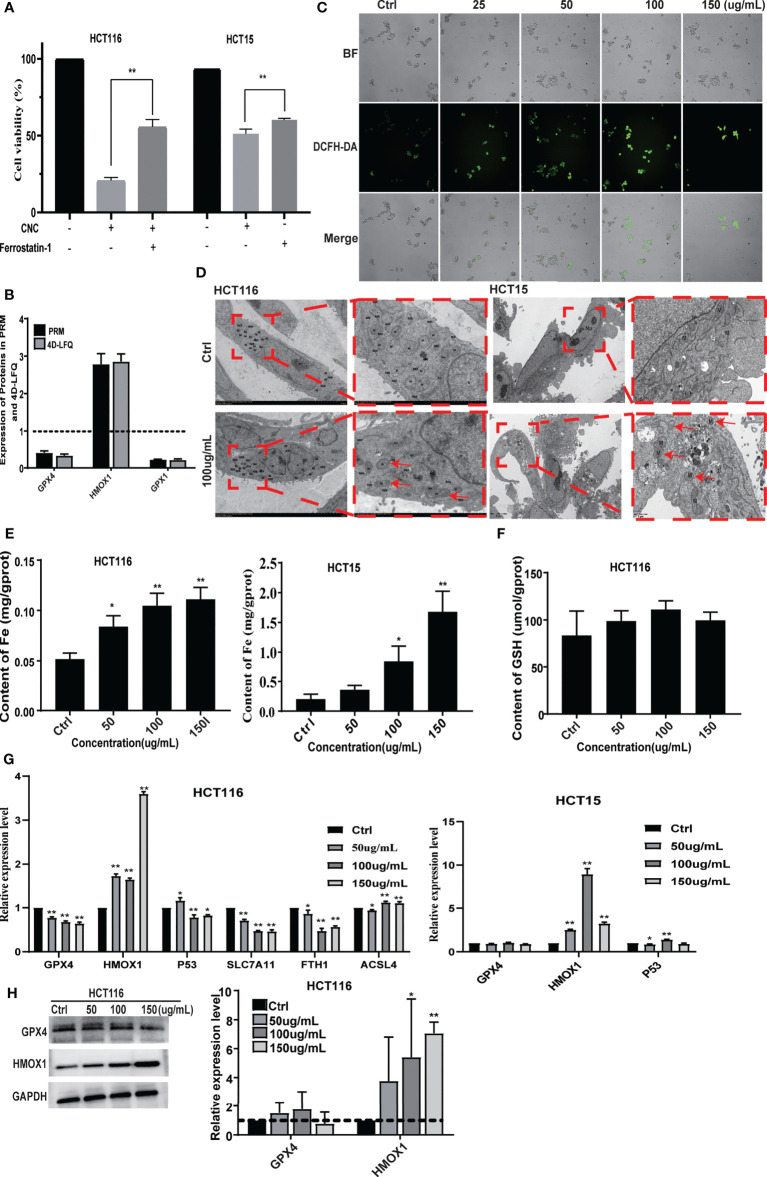
CNC extract induce cell death by promoting ferroptosis. **(A)** The ferrostatin-1 rescue experiment. **(B)** Ferroptosis-associated proteins quantification by mass spectrometry-based targeted proteomics (PRM). **(C)** The detection of HCT116 cellular ROS accumulation. **(D)** The TEM analysis of HCT116 and HCT15 cells morphology. **(E, F)** The detection of cellular Fe and GSH content. **(F)** Expression of relevant proteins at mRNA level were detected by q-PCR. **(G)** Expression of GPX4 and HMOX1 in HCT116 cells was measured by western blot. There were three duplicate samples in each experiment. Data are expressed as mean ± SD. Compared with control group: *p < 0.5, **p < 0.01.

PRM analysis is principal method used to verify the proteomics data that selects specific peptides or target peptides and quantifies them (17). We analyzed 15 of the 363 differentially expressed proteins using PRM instead of traditional western blots (see **Supplementary 5** **)**. First, according to the classic ferrostatin-1 rescue experiment, we found that the ferroptosis inhibitor ferrostatin-1 can effectively reverse the cell ferroptosis induced by CNC extract **(** **Figure 5A** **)**. Among them, there were three proteins associated with the ferroptosis pathway. 4D Label-free analysis showed that the ferroptosis-associated proteins GPX4 and GPX1 decreased, whereas HMOX1 increased **(** **Figure 5B** **)**. We observed the same results after the PRM analysis. Additionally, nuclear accumulation of ROS was increased in CNC extract-treated HCT116 cells **(** **Figure 5C** **)**. We hypothesized that ferroptosis may be highly related to CNC extract-induced cell death, and TEM revealed that HCT116 and HCT15 cells treated with CNC extract exhibited characteristic features of ferroptosis and the most significantly changed mitochondria was indicated by red arrows. **(** **Figure 5D** **)**, such as shrunken mitochondria with increased membrane density and the appearance of autophagy. While the iron content of HCT116 and HCT15 cells were significantly increased in the CNC extract group, there were no changes in HCT116 of GSH content **(** **Figures 5E, F** **)**. The result of no significant change in GSH content was consistent with the research results of other scholars, and they found the second class inhibits GPX4 without GSH depletion, such as RSL3, which inhibits GPX4 directly (18). In ferroptosis, GPX4 protects against iron- and oxygen-dependent lipid peroxidation by converting lipid peroxides into nontoxic lipids. Inhibition of GPX4 will lead to peroxide accumulation and the occurrence of ferroptosis (19). HMOX1 can catalyze the degradation of heme to Fe^2+^, biliverdin, and carbon monoxide. Excess Fe^2+^ promotes ferroptosis through the Fenton reaction (20). Other hallmarks of ferroptotic pathway, such as p53, SLC7A11, FTH1 and ACSL4 in HCT116 and HCT15 were determined. Notably, it was found that CNC extract could reduce mRNA expression levels of GPX4, SLC7A11 and FTH1 in HCT116. And CNC extract could increase mRNA expression levels of HMOX1, P53 and ACSL4 in HCT116. Also, CNC extract could increase mRNA expression levels of HMOX1 and P53 in HCT15, but had no significant effect on the expression of other hallmarks of ferroptotic pathway **(** **Figure 5G** **)**. Similar expression levels of HMOX1 protein were observed in western blot and R-PCR. As for GPX4, western blot and q-PCR showed the same results and down-regulated the expression of GPX4, but there was no significant difference in western blot **(** **Figure 5H** **)**. These results provide further evidence that CNC extract may inhibit the proliferation of colon cancer cells through ferroptosis-related pathways.

### CNC Extract Suppresses Tumor Growth and Regulates the Ferroptosis Pathway *In Vivo*

**Figure 6:**
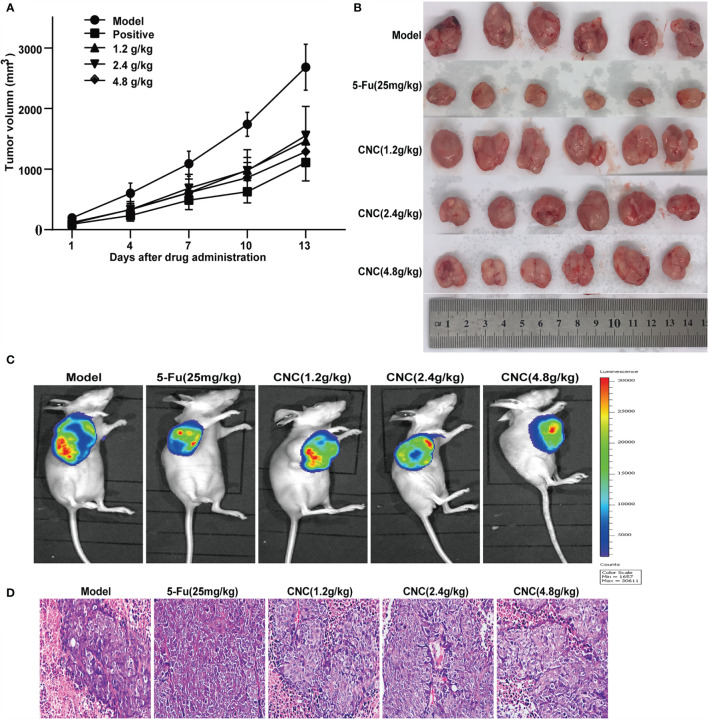
CNC extract inhibited tumor growth in human HCT116 xenograft nude mouse model. **(A, B)** Tumor growth curves and volume in CNC extract-treatment and model groups. **(C)** Bioluminescence imaging of HCT116 xenograft tumors in different groups at the end of experiments. **(D)** HE staining of tumor tissues. There were six nude mice in each group.

**Figure 7:**
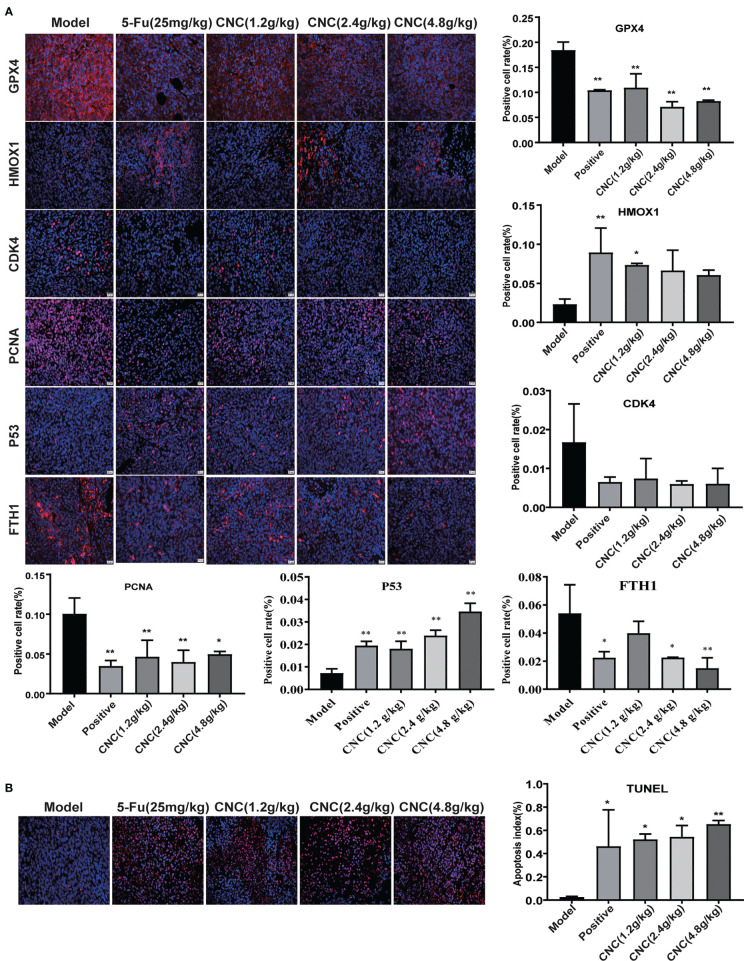
IF detection and TUNEL analysis in tumor tissues. **(A)** IF expression of GPX4, HMOX1, CDK4, PCNA, p53 and FTH1proteins in tumor tissue. **(B)** TUNEL analysis in tumor tissues. There were six nude mice in each group. Data are expressed as mean ± SD. Compared with model group: *p < 0.5, **p < 0.01.

After 14 consecutive days of treatment with CNC extract, xenografts of CNC extract-treated groups had smaller tumors compared to the model group **(** **Figures 6A, B** **)**. Bioluminescence imaging of HCT116 xenograft tumors in different groups showed consistent results for the measured tumor volume **(** **Figure 6C** **)**. Pathological sections of tumor tissues were also observed with hematoxylin and eosin staining **(** **Figure 6D** **)**. Immunofluorescence staining of ferroptosis- and cell proliferation-related markers (GPX4, FTH1, PCNA, and CDK4 positive ratio) decreased, while HMOX1 and p53 increased in the CNC extract-therapy group **(** **Figure 7A** **)**. Consistent with the *in vitro* experiments, TUNEL assays showed that apoptosis rates were significantly increased in the CNC extract-therapy group compared to the model group **(** **Figure 7B** **)**.

## Discussion

Colon cancer is a malignant tumor and the fourth leading cause of cancer-related deaths (21). Although progress has been made in diagnosis and treatment, the prognosis of the patients is still inferior (22). Recent years have seen the rapid rise and development of TCM. Compared with single-target chemical drugs with high toxicity, side effects, and poor efficacy, the clinical advantages of TCMs with multiple components, pathways, and targets are becoming more prominent (23–25). CNC is a TCM with a medicinal history of more than 2000 years. Modern pharmacological studies have suggested that CNC has a wide range of anti-tumor properties (26, 27), in liver cancer, lung cancer, esophageal cancer, and others. Here we studied the anti-colon cancer efficacy of CNC *in vivo* and *in vitro* to explore potential mechanisms underlying these effects. Our results suggest that CNC is a promising candidate for the prevention and treatment of colon cancer.

Ferroptosis, a form of regulated cell death identified by Stockwell and colleagues in 2012, is mediated by iron-dependent accumulation of lipid ROS (28). In recent years, studies have found that the mechanism and treatment of tumors are closely related to ferroptosis (29, 30). Our study provides the first evidence that CNC can regulate ferroptosis signaling pathways, thus inhibiting the proliferation of colon cancer cells *in vitro* and *in vivo*. Ferroptosis is mainly related to intracellular iron metabolism, lipid peroxide (ROS) content, and GPX4 activity. Iron transporters are negatively regulated by ferritin. Studies have shown significantly increased ferritin in tumor patients with inhibited iron efflux (31). The anti-tumor mechanism of dihydroartemisinin in the human hepatoma cell line HepG2 is to induce ferroptosis by increasing ROS and reducing GSH expression in cancer cells through a Fenton-like reaction (32). Here, we verified the ferroptosis pathway of CNC against colon cancer. TEM revealed typical morphological characteristics of ferroptosis in CNC-treated HCT116 and HCT15 cells, such as shrunken mitochondria with increased membrane density. And we also found that the ferroptosis inhibitor ferrostatin-1 can effectively reverse the cell ferroptosis induced by CNC extract. Measuring the intracellular contents of ROS, Fe, and GSH revealed that CNC significantly induced the accumulation of ROS in HCT116 cells and induced the accumulation of Fe in HCT116 and HCT15 cells and finally mediated ferroptosis. Furthermore, the key ferroptosis pathway proteins were verified *in vitro*. qPCR results showed that significantly decreased mRNA expression of GPX4, SLC7A11 and FTH1 in HCT116 cells, while that of HMOX1, P53 and ACSL4 were significantly increased. Also, we observed the increase mRNA expression levels of HMOX1 and P53 in HCT15, but had no significant effect on the expression of other hallmarks of ferroptotic pathway. These results were corroborated by western blot analyses. In the immunofluorescence experiments of tumor tissues in nude mice, GPX4 and FTH1 expression were down-regulated and HMOX1 and p53 were up-regulated in the CNC extract group compared to the model group. These results suggested that the anti-colon cancer effect of CNC may be related to inhibition of GPX4, FTH1 and promotion of HMOX1, p53 expression, which is similar to the mechanism by which artesunate induces ferroptosis in tumor cells (33).

Proteomics is the study pertaining to protein products encoded and expressed by genes and is a powerful tool for understanding the interactions of protein molecules in the human body (34). The proteomics of cells or bodies under normal conditions, diseases, and during drug treatment helps clarify the molecular mechanisms underlying the actions of macromolecules and facilitates the identification of new drug targets, drug development, and the drafting of clinical treatment guidelines (35). In our study, we combined 4D Label-free quantitative proteomics with PRM to analyze protein changes in HCT116 cells to find potential pathways and targets of CNC. The results showed that CNC could regulate apoptosis, cell cycle, ferroptosis, and other pathways of colon cancer cells, and we further detected ferroptosis pathway proteins. In addition to effects on ferroptosis, CNC was also found to promote apoptosis and block cell cycling, indicating that CNC may exert anti-tumor effects through multiple pathways.

The main chemical components of CNC are flavonoids, polysaccharides, saponins, volatile components, and trace elements (36). In our research, 116 CNC compounds were detected by UHPLC-MS. According to published literature, 10 of these compounds have been isolated from CNC, although their contents were not among the highest. The mass spectrum of CNC contains several high intensity peaks that have not been resolved, indicating that there may be other anti-tumor components in CNC. Further studies are needed to clarify the detailed biochemical and physiological mechanisms of action associated with the anti-tumor effects of CNC.

**Figure 8:**
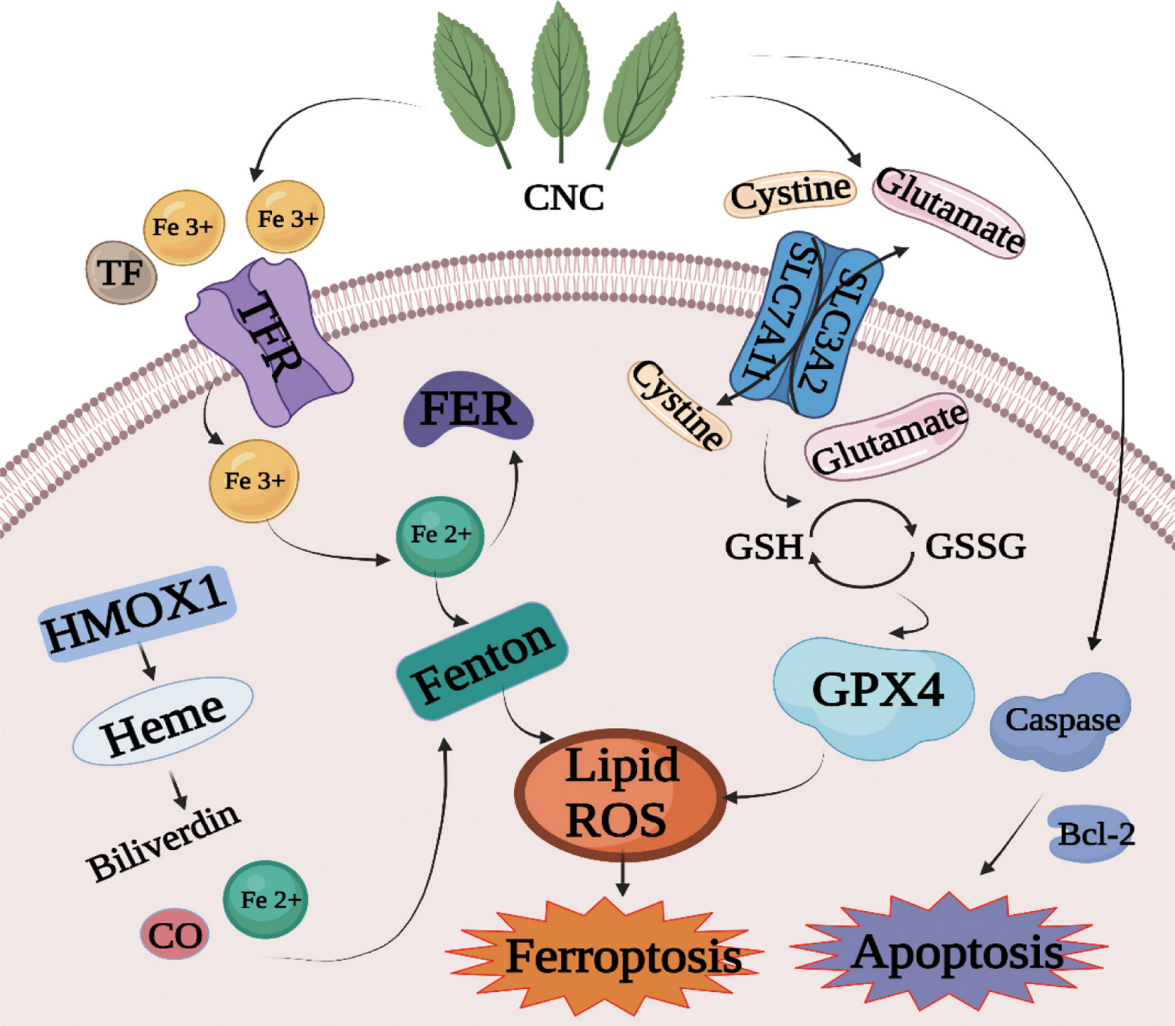
Mechanism diagram of cell death induced by CNC extract through the apoptosis and ferroptosis pathway.

In summary, we studied the chemical constituents, anti-tumor pharmacological effects, and mechanism of CNC. Flavonoids, polysaccharides, volatile components, and triterpenes were detected by UHPLC-MS. A combination of 4D Label-free quantitative proteomics and PRM technology was used to identify the potential targets and pathways underlying the anti-colon cancer effect of CNC. The results showed that CNC could regulate cell apoptosis, cell cycle, ferroptosis, and other pathways in colon cancer cells, indicating multi-pathway regulatory effects. We detected and verified components of the ferroptosis pathway and found that CNC regulates GPX4, HMOX1, SLC7A11, FTH1, p53 and ACSL4 protein expression to exert its anti-colon cancer effects. The potential mechanism by which CNC extract affects the apoptosis and ferroptosis pathway is shown in **Figure 8**. In conclusion, CNC is a promising candidate for the prevention and treatment of colon cancer.

## Data Availability Statement

The datasets presented in this study can be found in online repositories. The names of the repository/repositories and accession number(s) can be found below: https://www.jianguoyun.com/p/DTo3RE8Qh4fOCRio3P0D.

## Ethics Statement

The animal study was reviewed and approved by Animal Ethics Committee of the Guangxi University of Chinese Medicine, and all procedures were followed the relevant regulations and guidelines.

## Author Contributions

JD, EH and XH designed the study. YC and FZ drafted the manuscript. YC and FZ conducted experiments, and the other authors took part in literature collection and data analysis. All authors contributed to the article and approved the submitted version.

## Funding

This study was supported by grants from Guangxi Special Project for Innovation-Driven Development (AA18118049); Guangxi Science and Technology Plan Project (AD19110155); Guangxi Science and Technology Base and Talent Special Fund (GuiKe AD21075014).

## Conflict of Interest

The authors declare that the research was conducted in the absence of any commercial or financial relationships that could be construed as a potential conflict of interest.

## Publisher’s Note

All claims expressed in this article are solely those of the authors and do not necessarily represent those of their affiliated organizations, or those of the publisher, the editors and the reviewers. Any product that may be evaluated in this article, or claim that may be made by its manufacturer, is not guaranteed or endorsed by the publisher.
