# Supplementary material for: Proteome Analysis of Camellia nitidissima Chi Revealed Its Role in Colon Cancer Through the Apoptosis and Ferroptosis Pathway
Source: Front Oncol. 2021 Nov 9;11:727130. doi: 10.3389/fonc.2021.727130 (PMC8630681; doi:10.3389/fonc.2021.727130)
Supplement: Supplementary file 1 [file DataSheet_1.docx]

Supplementary Material

**Supplement 1**

HCT116 cells were seeded into 15-cm dishes. After 24 h of culture, cells were treated with CNC extract (100 μg/mL) and DMSO for 48 h, respectively (3×biological replicates). Samples were sonicated three times on ice using a high intensity ultrasonic processor (Scientz) in lysis buffer (1% Triton X-100, 1% protease inhibitor cocktail). The remaining debris was removed by centrifugation at 12,000 g at 4℃ for 10 min. Finally, the supernatant was collected and the protein concentration was determined with BCA kit according to the manufacturer’s instructions. Next, The same amount of each sample protein was taken for protein digestion, and the volume was adjusted to the same with lysate. The protein sample was added with 1 volume of pre-cooled acetone, vortexed to mix, and added with 4 volumes of pre-cooled acetone, and then precipitated at -20℃ for 2 h. The precipitate was collected by centrifugation at 5500 g for 5 min at 4℃. The precipitated protein was washed with pre-cooled acetone for 3 times and finally acetone was removed by drying in a fume cupboard. The protein sample was then added 200 mM TEAB and ultrasonically dispersed. Finally, Trypsin was added at 1:50 trypsin-to-protein mass ratio for the first digestion overnight. The sample was reduced with 5 mM dithiothreitol for 60 min at 37℃ and alkylated with 11 mM iodoacetamide for 45 min at room temperature in darkness.

The tryptic peptides were dissolved in solvent A (0.1% formic acid, 2% acetonitrile in water), directly loaded onto a home-made reversed-phase analytical column (25-cm length, 100 μm i.d.). Peptides were separated with a gradient from 6% to 24% solvent B (0.1% formic acid in acetonitrile) over 70 min, 24% to 35% in 14 min and climbing to 80% in 3 min then holding at 80% for the last 3 min, all at a constant flow rate of 450 nL/min on a nanoElute UHPLC system (Bruker Daltonics). The peptides were subjected to Capillary source followed by the timsTOF Pro (Bruker Daltonics) mass spectrometry. The electrospray voltage applied was 1.75 kV. Precursors and fragments were analyzed at the TOF detector, with a MS/MS scan range from 100 to 1700 m/z. The timsTOF Pro was operated in parallel accumulation serial fragmentation (PASEF) mode. Precursors with charge states 0 to 5 were selected for fragmentation, and 10 PASEF-MS/MS scans were acquired per cycle. The dynamic exclusion was set to 30 s.

**Supplement 2**

Tandem mass spectra were searched against the Homo sapiens 9606 SwissProt database (20366 entries) concatenated with reverse decoy database. Trypsin/P was specified as cleavage enzyme allowing up to 2 missing cleavages. The mass tolerance for precursor ions was set as 20 ppm in First search and 20 ppm in Main search, and the mass tolerance for fragment ions was set as 20 ppm. Carbamidomethyl on Cys was specified as fixed modification, and acetylation on protein N-terminal and oxidation on Met were specified as variable modifications. FDR was adjusted to < 1%.

All identified proteins were subjected to annotation methods, including Gene Ontology (GO) annotation derived from the UniProt-GOA database ( http://www.ebi.ac.uk/GOA/) in terms of biological processes (BPs), cellular components (CCs), and molecular functions (MFs). And KEGG pathway annotation based on Kyoto Encyclopedia of Genes and Genomes (KEGG) database. For differentially expressed proteins (Ds to identify enrEPs), (fold-change > 1.5 or < 0.67), functional enrichment analysis was performed based on GO (biological process, cellular compartment and molecular function) and KEGG databaseiched terms by a two-tailed Fisher’s exact test. The terms with a corrected p-value < 0.05 was considered significant.

**Supplement 4**

**
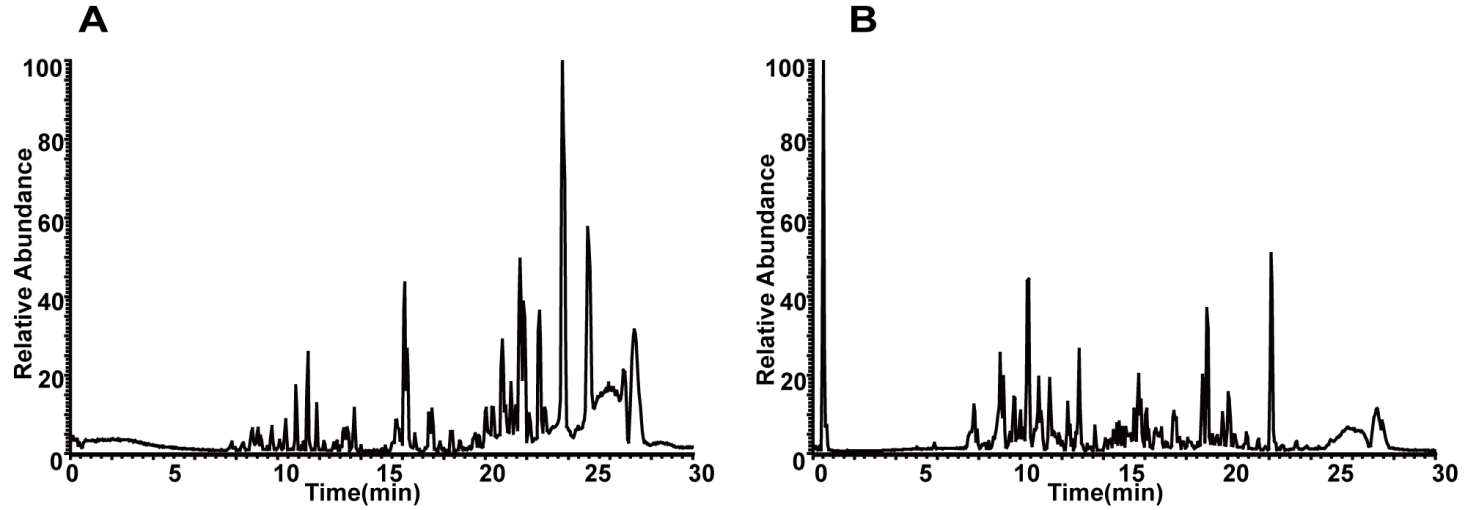
**

**Component analysis of CNC extract.** (A) The base peak chromatogram of CNC extract by UHPLC-MS in positive mode. (B) The base peak chromatogram of CNC extract by UHPLC-MS in negative mode.

**Supplement 5**

**Protein quantification of PRM**

| Protein Accession | Gene | Y1/C1 Ratio | Y2/C2 Ratio | Y3/C3 Ratio | Y/C Ratio | Y/C P value |
| --- | --- | --- | --- | --- | --- | --- |
| Q9P0J0 | NDUFA13 | 0.40 | 0.40 | 0.44 | 0.42 | 1.38E-05 |
| Q15392 | DHCR24 | 2.54 | 2.62 | 2.82 | 2.66 | 4.42E-05 |
| O43504 | LAMTOR5 | 0.77 | 1.05 | 0.80 | 0.86 | 1.40E-01 |
| P07203 | GPX1 | 0.22 | 0.23 | 0.23 | 0.23 | 1.65E-06 |
| P07858 | CTSB | 0.46 | 0.36 | 0.36 | 0.39 | 1.80E-03 |
| P09601 | HMOX1 | 2.64 | 2.61 | 3.11 | 2.78 | 3.22E-05 |
| Q9NVS2 | MRPS18A | 0.86 | 0.86 | 0.81 | 0.84 | 3.31E-03 |
| Q16822 | PCK2 | 0.54 | 0.54 | 0.56 | 0.55 | 3.40E-06 |
| Q13501 | SQSTM1 | 4.66 | 4.97 | 4.60 | 4.75 | 2.43E-05 |
| Q53EL6 | PDCD4 | 0.68 | 0.69 | 0.65 | 0.67 | 1.77E-04 |
| Q00535 | CDK5 | 1.04 | 1.11 | 0.87 | 1.00 | 9.65E-01 |
| Q9ULV4 | CORO1C | 1.40 | 1.26 | 1.44 | 1.36 | 1.74E-03 |
| P50613 | CDK7 | 1.11 | 1.50 | 2.17 | 1.52 | 4.06E-02 |
| Q13751 | LAMB3 | 3.16 | 3.19 | 3.83 | 3.39 | 4.81E-04 |
| P36969 | GPX4 | 0.37 | 0.46 | 0.38 | 0.40 | 5.84E-05 |
